# Anti-Inflammatory Role of the cAMP Effectors Epac and PKA: Implications in Chronic Obstructive Pulmonary Disease

**DOI:** 10.1371/journal.pone.0031574

**Published:** 2012-02-21

**Authors:** Anouk Oldenburger, Sara S. Roscioni, Esther Jansen, Mark H. Menzen, Andrew J. Halayko, Wim Timens, Herman Meurs, Harm Maarsingh, Martina Schmidt

**Affiliations:** 1 Department of Molecular Pharmacology, University of Groningen, Groningen, The Netherlands; 2 Department of Physiology and Internal Medicine, University of Manitoba, Winnipeg, Manitoba, Canada; 3 Department of Pathology and Medical Biology, University Medical Center Groningen and University of Groningen, Groningen, The Netherlands; 4 Groningen Research Institute for Asthma and COPD, Groningen, The Netherlands; Helmholtz Zentrum München/Ludwig-Maximilians-University Munich, Germany

## Abstract

Cigarette smoke-induced release of pro-inflammatory cytokines including interleukin-8 (IL-8) from inflammatory as well as structural cells in the airways, including airway smooth muscle (ASM) cells, may contribute to the development of chronic obstructive pulmonary disease (COPD). Despite the wide use of pharmacological treatment aimed at increasing intracellular levels of the endogenous suppressor cyclic AMP (cAMP), little is known about its exact mechanism of action. We report here that next to the β_2_-agonist fenoterol, direct and specific activation of either exchange protein directly activated by cAMP (Epac) or protein kinase A (PKA) reduced cigarette smoke extract (CSE)-induced IL-8 mRNA expression and protein release by human ASM cells. CSE-induced IκBα-degradation and p65 nuclear translocation, processes that were primarily reversed by Epac activation. Further, CSE increased extracellular signal-regulated kinase (ERK) phosphorylation, which was selectively reduced by PKA activation. CSE decreased Epac1 expression, but did not affect Epac2 and PKA expression. Importantly, Epac1 expression was also reduced in lung tissue from COPD patients. In conclusion, Epac and PKA decrease CSE-induced IL-8 release by human ASM cells via inhibition of NF-κB and ERK, respectively, pointing at these cAMP effectors as potential targets for anti-inflammatory therapy in COPD. However, cigarette smoke exposure may reduce anti-inflammatory effects of cAMP elevating agents via down-regulation of Epac1.

## Introduction

Chronic obstructive pulmonary disease (COPD) is a chronic inflammatory disorder characterized by infiltration of inflammatory cells into the airways. Cigarette smoke-induced inflammation is a main player in the development of COPD [1]. Neutrophils are an important component of the inflammation as they release inflammatory mediators and proteinases, which are believed to play a role in the pathogenesis of COPD [2], [3]. Moreover, neutrophil number has been associated with COPD severity [2], [4] and exacerbation frequency [5]. Interleukin-8 (IL-8) is a potent neutrophil chemoattractant and activator [2]; its abundance correlates with neutrophil counts in COPD [6], and is increased in sputum [7], in broncho-alveolar lavage fluid [8] and in the bronchiolar epithelium from COPD patients [9]. Furthermore, mRNA expression of IL-8 in bronchial biopsies correlates with COPD progression [10]. Cigarette smoke induces release of IL-8 from inflammatory cells [11], [12] and structural cells in the lung [9], [13], including airway smooth muscle (ASM) cells [14], [15]. *In vitro*, IL-8 release by cigarette smoke extract (CSE) involves activation of nuclear factor κappa B (NF-κB) [12], [14] and extracellular signal-regulated kinase (ERK) [11], [14]. Activation of NF-κB requires phosphorylation and degradation of IκBα and subsequent nuclear translocation of the NF-κB subunit p65 [16], [17], whereas ERK activation occurs via phosphorylation and subsequent nuclear translocation [18].

Currently, the most effective intervention for COPD is smoking cessation [19], but no preventive or curative pharmacological treatment exists. Despite their immuno-suppressive effects in asthmatics, corticosteroids do not exhibit significant anti-inflammatory properties in patients with COPD [20]–[22], partly due to the different types of inflammation involved. COPD therapy is primarily aimed on symptomatic treatment, which includes the use of bronchodilator drugs such as inhaled β_2_-agonists that increase intracellular cyclic adenosine monophosphate (cAMP) in ASM [23]. Of note, β_2_-agonists can inhibit cytokine release *in vitro* [24]–[26], but evidence of their anti-inflammatory effectiveness of β_2_-agonists *in vivo* is lacking, which may be due to the β_2_-adrenergic receptor desensitization in both airway inflammatory and structural cells [27], [28]. Hence, activation of post-β_2_-adrenergic receptor mechanisms could be advantageous to maintain the beneficial effects of β_2_-agonists without the risk of receptor desensitization.

Among the structural cells in the airways, ASM cells represent a promising therapeutic target in chronic obstructive lung disease, due to their multifunctional behavior that subserves bronchoconstriction, wound healing and local inflammation [29]. Moreover, ASM release IL-8 [30] and express G_s_ protein-coupled β_2_-adrenergic receptors that couple with the cAMP effectors [23] protein kinase A (PKA) and exchange proteins activated by cAMP (Epac). Recently, we have shown that PKA and Epac modulate IL-8 release in ASM cells via an ERK-dependent mechanism [31]. Although the contribution of the different cAMP effectors was not studied, cAMP elevation by the β_2_-agonist salmeterol inhibited CSE-induced IL-8 release by human neutrophils [11]. We investigate here the modulatory role of Epac and PKA in CSE-induced IL-8 release by ASM cells and the underlying molecular mechanisms. We report that Epac and PKA exert their anti-inflammatory properties upon the inhibition of NF-κB and ERK, respectively. We also demonstrate that CSE reduced specifically Epac1 protein expression, both *in vitro* and in COPD patients.

## Results

### cAMP signalling attenuates CSE-induced IL-8 release from human ASM cells

**Figure 1:**
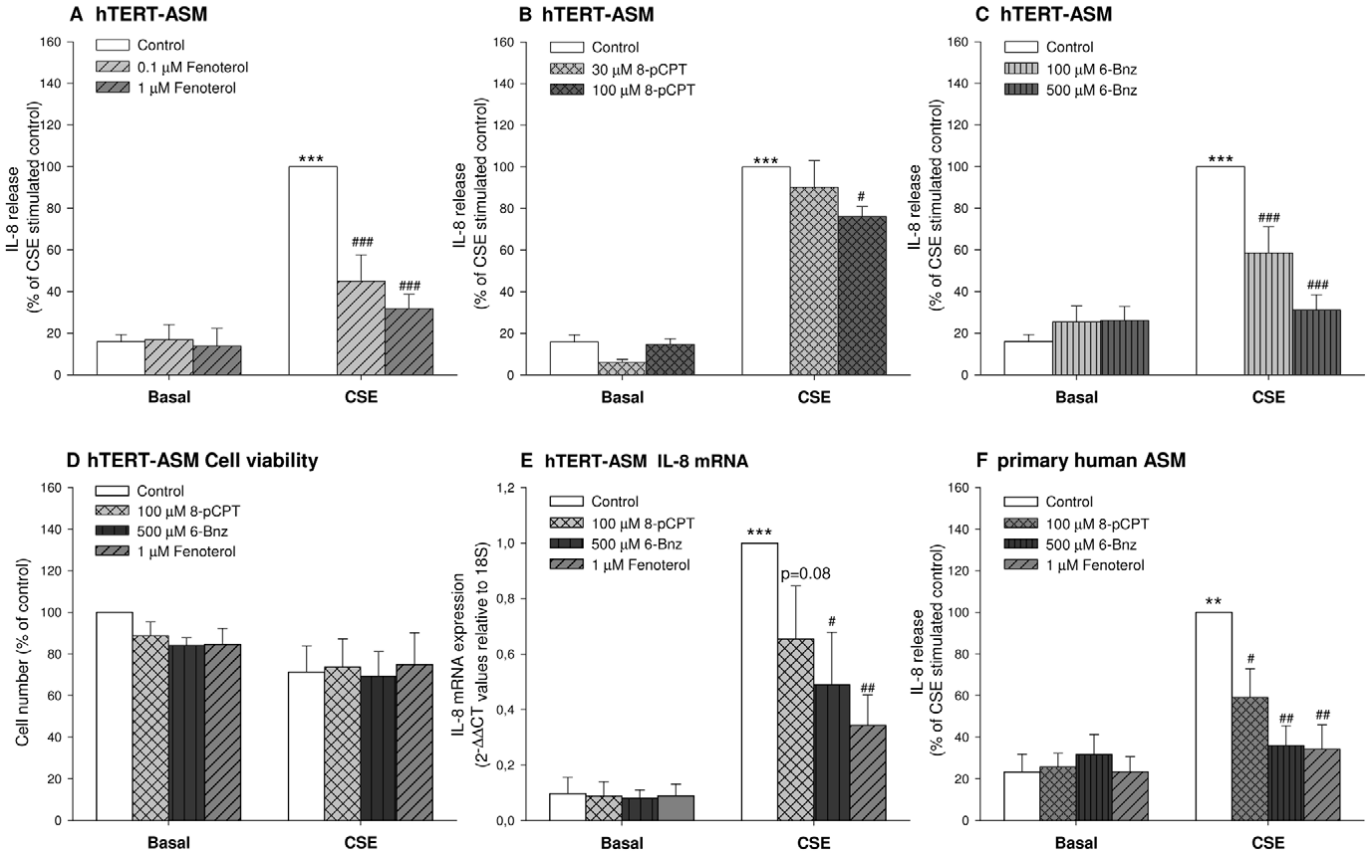
Fenoterol, 6-Bnz-cAMP and 8-pPCT-2′-*O*-Me-cAMP reduce CSE-induced IL-8 release from human ASM cells. hTERT ASM cells (A, B and C) and human primary ASM cells (F) were stimulated with 15% CSE in the absence or presence of fenoterol (0.1 or 1 µM), 6-Bnz-cAMP (100 µM or 500 µM) or 8-pCPT-2′-*O*-Me-cAMP (30 or 100 µM) for 24 hrs. Basal IL-8 release was 109,4±33,2 pg/ml in hTERT ASM cells and 7,5±4,7 in primary human ASM cells. Cells stimulated with 15% CSE for 6 hrs showed an increase in IL-8 mRNA expression which was reduced by fenoterol (1 µM), 6-Bnz-cAMP (500 µM) or 8-pCPT-2′-O-Me-cAMP (100 µM) (E). CSE, Fenoterol, 6-Bnz-cAMP and 8-pPCT-2′-*O*-Me-cAMP did not affect cell viability (D). Results are represented as means ± SEM of 6–22 separate experiments. Statistical analysis was performed by one-way ANOVA followed by a Newman-Keuls post-hoc test. ^***^ *P*<0.001,^ **^ *P*<0.01 compared to basal control; ^#^ *P*<0.05, ^##^ *P*<0.01. ^###^ *P*<0.001 compared to CSE-stimulated control.

Stimulation of hTERT-ASM cells with 15% CSE for 24 hrs significantly increased basal IL-8 release of approximately 7-fold (Fig. 1A–C), without affecting cell viability (Fig. 1D).

The CSE-induced IL-8 release was almost fully inhibited by co-treatment with the β_2_-agonist fenoterol (0.1 and 1 µM; *P*<0.001 both; Fig. 1A), in a concentration-dependent manner. To study the role of individual cAMP effectors Epac and PKA in the inhibition of CSE-induced release of IL-8, we applied the selective PKA activator 6-Bnz-cAMP (100 and 500 µM) and the selective Epac activator 8-pCPT-2′-*O*-Me-cAMP (30 and 100 µM). Treatment with 6-Bnz-cAMP concentration-dependently and fully inhibited CSE-induced IL-8 release (*P*<0.001; Fig. 1C). Only the highest concentration of 8-pCPT-2′-*O*-Me-cAMP (100 µM) inhibited IL-8 release, albeit to a lesser degree as compared to activation of PKA (*P*<0.05; Fig. 1B). None of the stimuli significantly altered basal IL-8 levels, in agreement with our previous observations [31] (Fig. 1A–C). Treatment with 8-pCPT-2′-*O*-Me-cAMP (100 µM), 6-Bnz-cAMP (500 µM) and fenoterol (1 µM) did not affect cell viability (Fig. 1D). Specific activation of Epac or of PKA did not affect the protein expression of these cAMP effectors either (not shown).

Analysis of IL-8 mRNA expression in hTERT-ASM showed that the effects on IL-8 release were reflected on the level of IL-8 gene transcription. Thus, the IL-8 mRNA expression was increased 6 hrs after stimulation with CSE (*P*<0.001). Treatment with 6-Bnz-cAMP (500 µM) and fenoterol (1 µM) significantly reduced the CSE-induced IL-8 mRNA expression (*P*<0.001 both), whereas a trend (*P* = 0.08) towards a reduction by 8-pCPT-2′-*O*-Me-cAMP (100 µM) was observed (Fig. 1E).

Similar to the effects in the immortalized cells, treatment with 15% CSE significantly enhanced IL-8 release of approximately 9-fold in *primary* human ASM cells (Fig. 1F). Co-stimulation with fenoterol (1 µM), 6-Bnz-cAMP (500 µM) and 8-pCPT-2′-*O*-Me-cAMP (100 µM) also reduced CSE-induced IL-8 release from primary human ASM cells (*P*<0.05, *P*<0.01), without affecting basal IL-8 levels (Fig. 1F).

**Figure 2:**
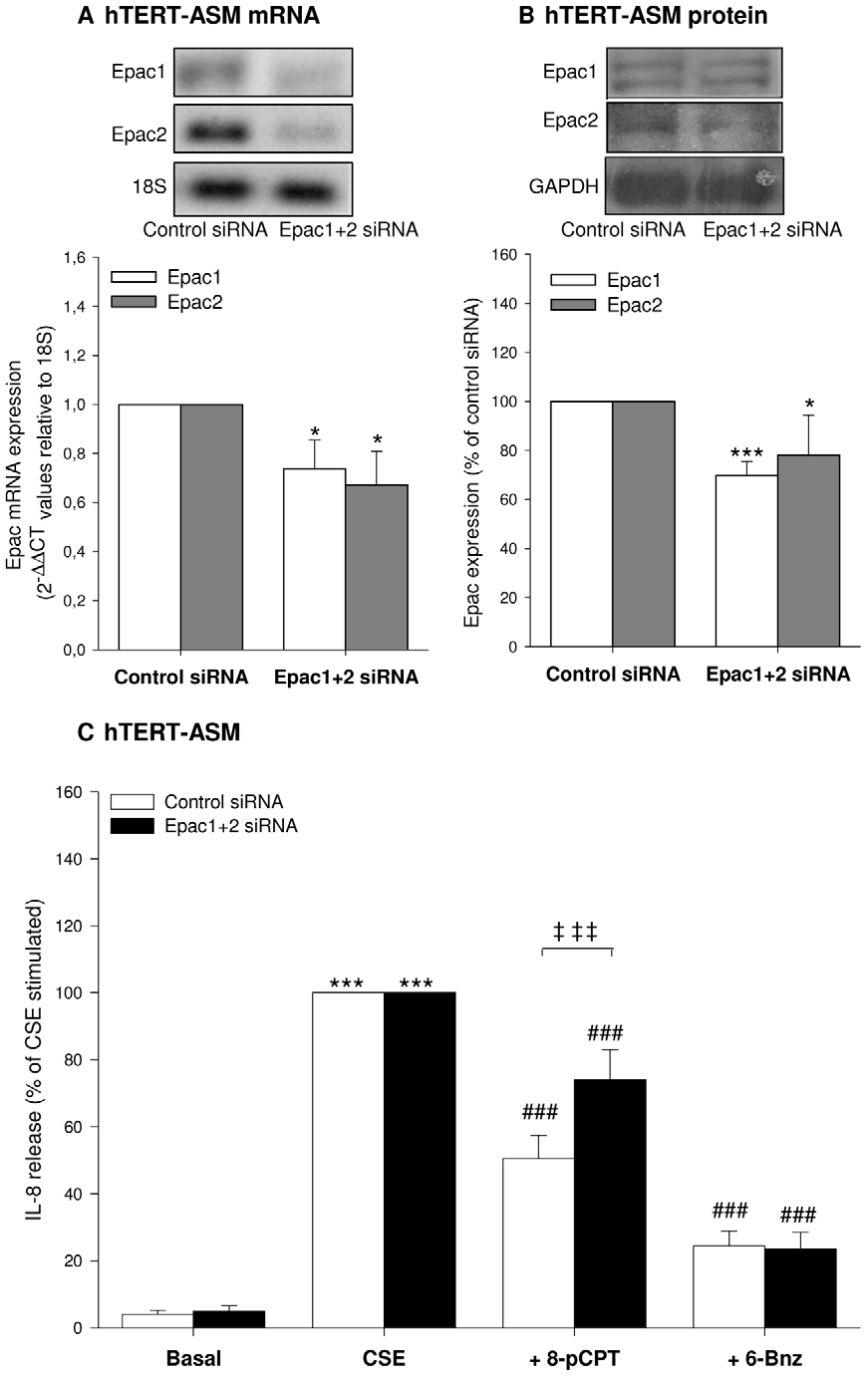
Epac knock-down attenuates the inhibitory effect of 8-pCPT-2′-*O*-Me-cAMP on CSE-induced IL-8 release. Epac1 and Epac2 mRNA and protein levels were analyzed after co-transfection with Epac1 and Epac2 siRNA or after transfection with control siRNA, resulting in a significant downregulation of both Epac1 and 2 mRNA (A) and protein (B) compared to control siRNA. Transfected hTERT-ASM cells were stimulated without (basal) or with 15% CSE in the absence or presence of either 500 µM 6-Bnz-cAMP or 100 µM 8-pCPT-2′-*O*-Me-cAMP for 24 hrs (C). Data are presented as means±SEM of 4–8 separate experiments. Statistical analysis was performed by one-way ANOVA followed by a Dunnett post-hoc test. *P<0.05, ^***^ *P*<0.001 compared to basal. ^#^ *P*<0.05; ^###^ *P*<0.001 compared to CSE. ^‡‡^ *P*<0.01, ^‡‡‡^ *P*<0.001.

**Figure 3:**
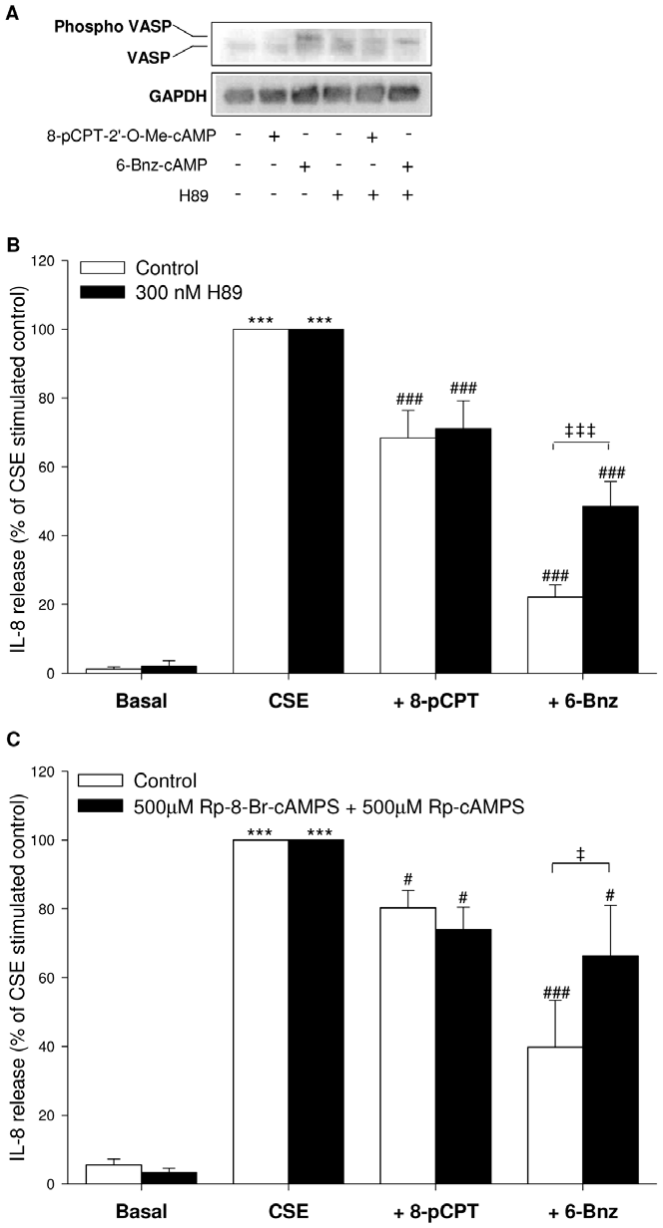
Inhibition of PKA attenuates the effect of 6-Bnz-cAMP on CSE-induced IL-8 release. Phosphorylation of VASP in hTERT-ASM cells treated with 100 µM 8-pCPT-2′-*O*-Me-cAMP or 500 µM 6-Bnz-cAMP in the absence or presence of the PKA inhibitor H89 (300 nM) was analysed by using an antibody, which recognizes both the phosphorylated VASP (phospho-VASP) and the non phosphorylated VASP (VASP) (A). VASP was normalized to GAPDH. Representative immunoblots of 3 experiments are shown. hTERT-ASM were pre-treated without (white bars) or with (black bars) 300 nM H89 (B) or 500 µM of both Rp-8-Br-cAMPS and Rp-cAMPS (C) for 30 min before stimulation with 15% CSE, 100 µM 8-pCPT-2′-*O*-Me-cAMP, 500 µM 6-Bnz-cAMP or their combinations. Data are presented as means±SEM of 3–9 separate experiments. Statistical analysis was performed by one-way ANOVA followed by a Newman-Keuls post-hoc test. ^***^ *P*<0.001 compared to basal control. ^#^ *P*<0.05, ^###^ *P*<0.001 compared to CSE. ^‡^ *P*<0.05, ^‡‡‡^ *P*<0.001.

The specific effects of the two cAMP effectors Epac and PKA on IL-8 release by hTERT-ASM cells were validated using different experimental approaches. siRNA probes against Epac1 and Epac2 were used to silence Epac [31]. Efficiency of transfection was evaluated by qPCR (Fig. 2A) and by western blot analysis (Fig. 2B). The mRNA and protein expression of Epac1 and Epac2 were both significantly down-regulated after co-transfection with Epac1 and Epac2 siRNA compared to control siRNA treatment (Fig. 2A–B). Treatment with siRNA for either Epac1 or Epac2 only slightly and non significantly reduced the inhibitory effect of 8-pCPT-2′-*O*-Me-cAMP on CSE-induced IL-8 release (not shown). However, simultaneous knock-down of both Epac1 and Epac2 mRNA and protein (Fig. 2C), effectively impaired the inhibitory effect of 8-pCPT-2′-*O*-Me-cAMP on CSE-induced IL-8 release from hTERT-ASM cells by about 50% (*P*<0.001, Fig. 2C), leaving the basal and CSE-induced IL-8 release unaffected (not shown). As expected, the effect of the PKA activator 6-Bnz-cAMP was not affected by the siRNA silencing of Epac1 and Epac2 (Fig. 2C). The specificity of the cAMP analogs was confirmed by evaluation of the phosphorylation of the PKA-specific substrate VASP (vasodilator-activated phosphoprotein). Thus, PKA activation with 6-Bnz-cAMP, but not Epac activation with 8-pCPT-2′-*O*-Me-cAMP, induced VASP phosphorylation, an effect that could be reduced by the PKA inhibitor H89 (300 nM; Fig. 3A). Moreover, the inhibitory effect of 6-Bnz-cAMP, but not 8-pCPT-2′-*O*-Me-cAMP, on CSE-induced IL-8 release was largely reduced by H89 (*P*<0.001, Fig. 3B) and by the combination of specific PKA inhibitors [32] Rp-8-Br-cAMP and Rp-cAMPS (500 µM, each *P*<0.01, Fig. 3C). None of the inhibitors altered Epac-mediated effects on CSE-induced IL-8 release (Fig. 3B–C).

### Role of NF-κB in CSE-induced IL-8 release

**Figure 4:**
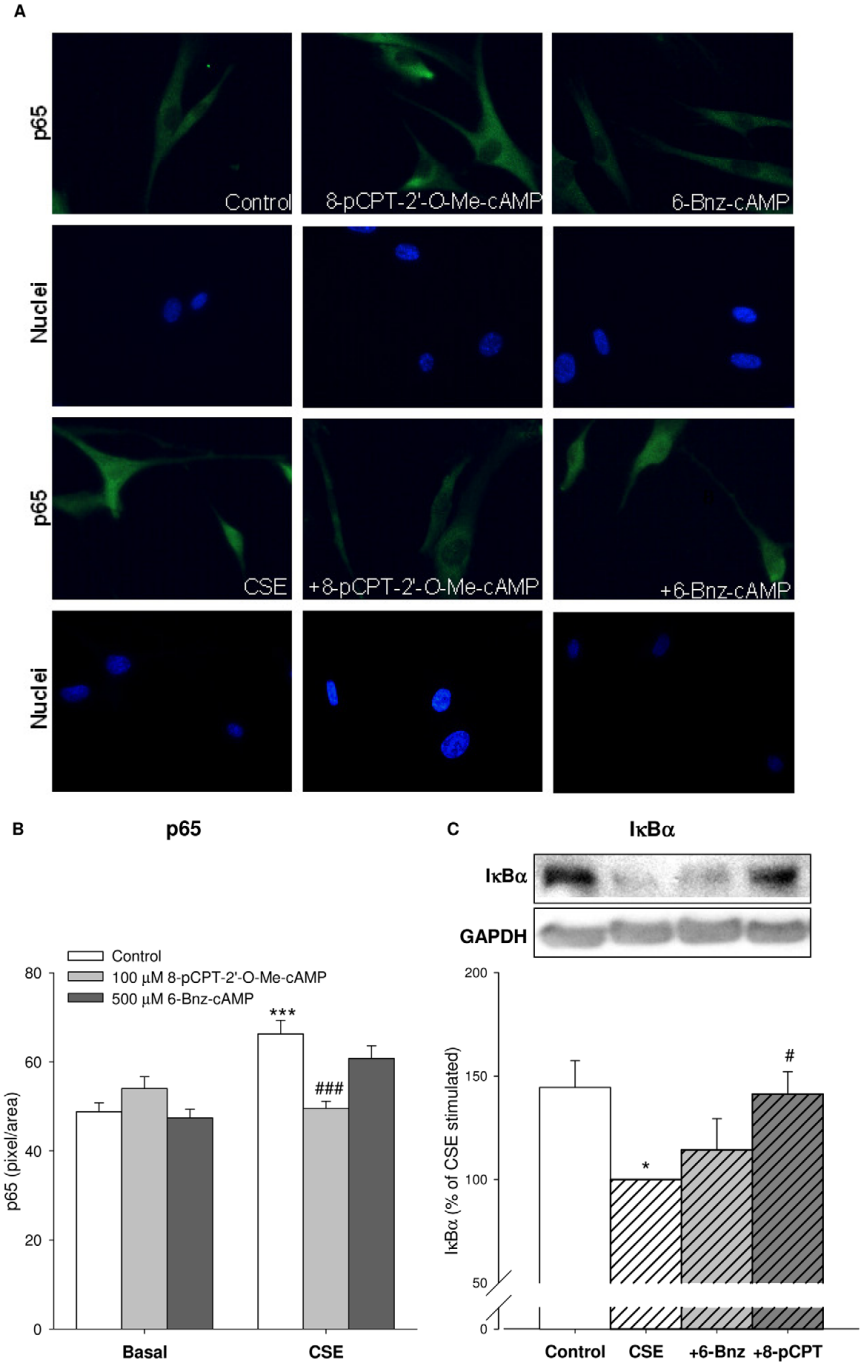
8-pCPT-2′-*O*-Me-cAMP prevents CSE-induced breakdown of IκBα and p65 nuclear translocation. p65 nuclear translocation was determined by immunofluorescence using p65 antibodies on hTERT-ASM cells stimulated without (control) or with 15% CSE for 2 hrs, alone or in combination with 100 µM 8-pCPT-2′-*O*-Me-cAMP or 500 µM 6-Bnz-cAMP. Representative results of 3 separate experiments are shown (A) with the quantification of p65 nuclear staining (B). hTERT-ASM cells were treated with 15% CSE, 100 µM 8-pCPT-2′-*O*-Me-cAMP, 500 µM 6-Bnz-cAMP or their combinations for 1 hr. Cells were lysed and IκBα levels were determined by Western blot analysis (C). Bands were normalized to GAPDH. Representative immunoblots of IκBα and GAPDH are shown. Data are presented as means±SEM of 6–7 separate experiments. Statistical analysis was performed by one-way ANOVA followed by a Dunnett post-hoc test. ^*^ *P*<0.05, ^***^ *P*<0.001 compared to basal control. ^#^ *P*<0.05, ^###^ *P*<0.001 compared to CSE.

NF-κB activation has been shown to be crucial for CSE-induced IL-8 production [11], [14]. In line, the IKK-2 inhibitor SC514 (50 µM) significantly reduced CSE-induced IL-8 release from hTERT ASM cells by >50% (*P*<0.001) (not shown), confirming the importance of the NF-κB pathway. Accordingly, treatment of hTERT-ASM cells with CSE for 2 hrs increased nuclear staining of the NF-κB subunit p65 (*P*<0.001, Fig. 4A–B), indicating increased translocation of the transcriptionally active NF-κB subunit from the cytosol to the nucleus. The Epac activator 8-pCPT-2′-*O*-Me-cAMP and the PKA activator 6-Bnz-cAMP did not affect basal p65 cellular localization (Fig. 4A). Importantly, p65 nuclear translocation by CSE was significantly inhibited by 8-pCPT-2′-*O*-Me-cAMP (*P*<0.001) (Fig. 4A–B). p65 nuclear translocation was preceded by loss (degradation) of the NF-κB inhibitory protein IκBα after 1 hr treatment with CSE (*P*<0.05, Fig. 4C). This response was largely prevented by the Epac activator (*P*<0.05 Fig. 4C), and only slightly upon PKA activation by 6-Bnz-cAMP (Fig. 4C). Taken together, these findings indicate that stabilization of the IκBα-p65 complex underlies the inhibitory effect of Epac on NF-κB activation.

### Role of ERK in CSE-induced IL-8 release

**Figure 5:**
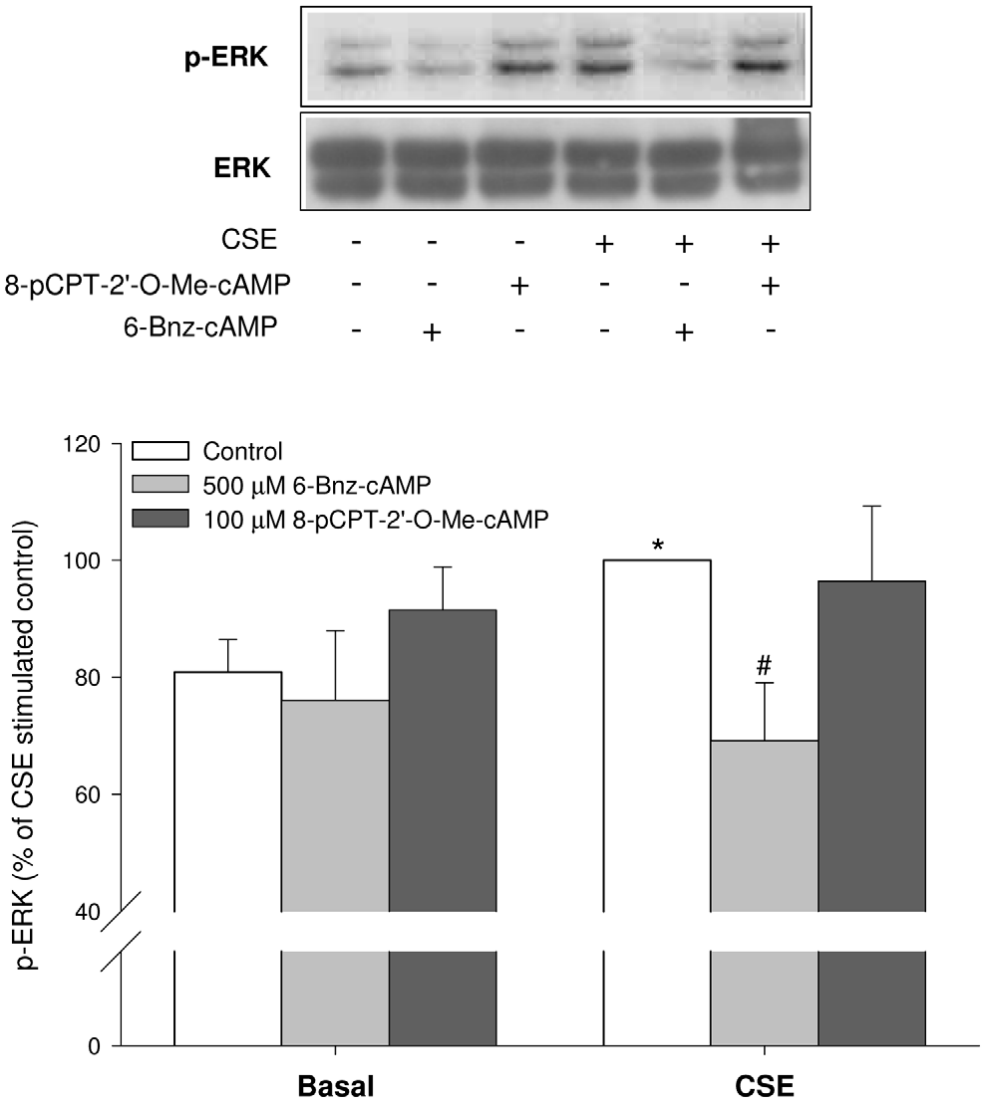
6-Bnz-cAMP prevents CSE-induced ERK phosphorylation. hTERT-ASM cells were lysed after being stimulated with 15% CSE, 100 µM 8-pCPT-2′-*O*-Me-cAMP, 500 µM 6-Bnz-cAMP or their combinations for 1 hr followed by Western blot analysis of phospho-ERK (p-ERK). Total ERK (ERK) was used as a loading control. Representative immunoblots of p-ERK and ERK are shown. Data are presented as means±SEM of 7–8 separate experiments. Statistical analysis was performed by one-way ANOVA followed by a Dunnett post-hoc test. ^*^ *P*<0.05 compared to basal control. ^#^ *P*<0.05 compared to CSE.

In line with previous studies from our group [14], [33], CSE induced a significant increase in ERK phosphorylation in hTERT-ASM cells after 1 hr of stimulation (*P*<0.05, Fig. 5). The importance of the ERK pathway was supported by the effect of MEK inhibitor U0126 (3 µM), which reduced CSE-induced IL-8 release by about 50% (*P*<0.001) (not shown). Importantly, the CSE-induced ERK activation was completely normalized by the PKA activator 6-Bnz-cAMP (*P*<0.05, Fig. 5) whereas activation of Epac had no effect (Fig. 5). These findings clearly reveal differential effects of PKA and Epac on CSE-induced ERK activation.

### Modulation of Epac expression by CSE

**Figure 6:**
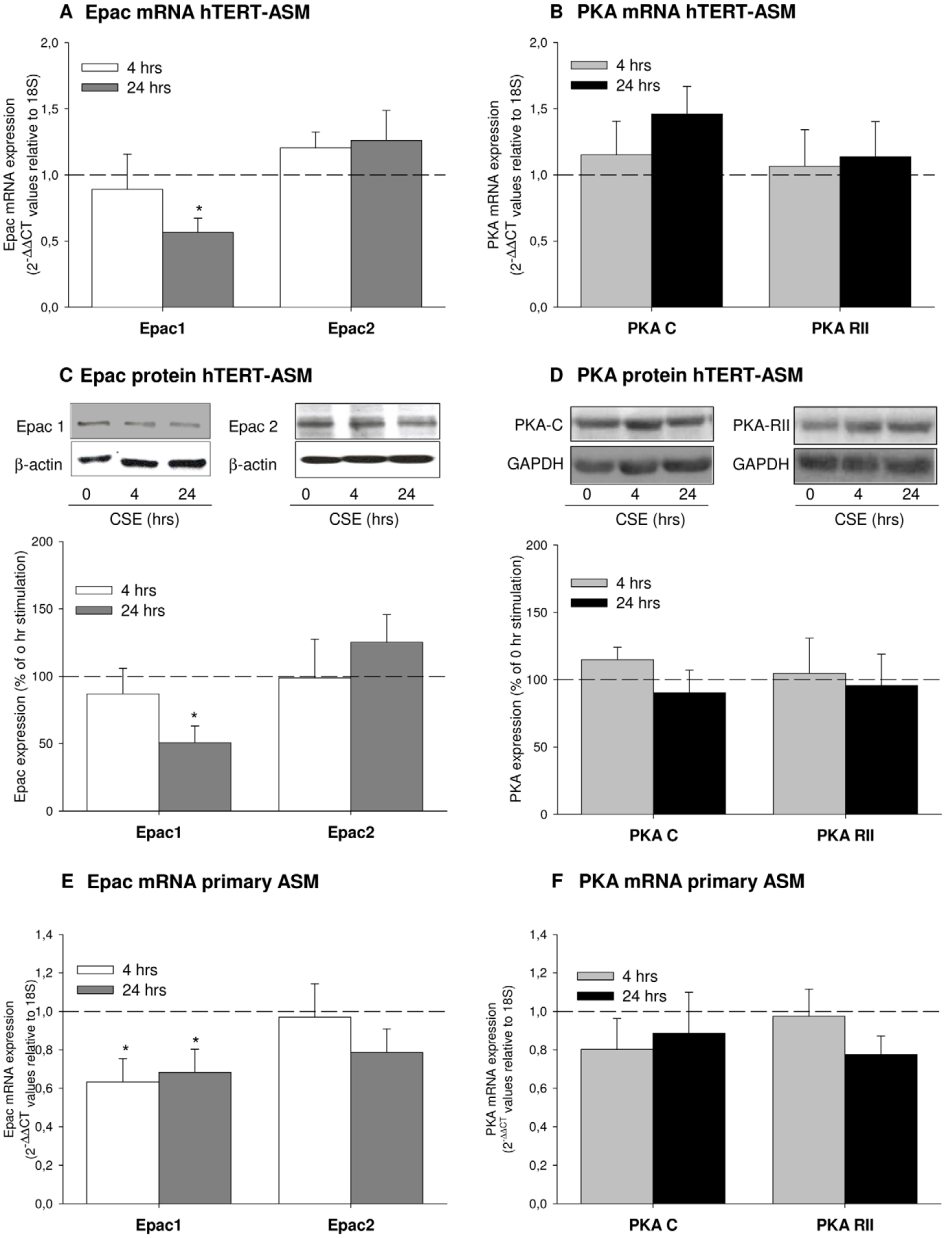
Epac1 is down-regulated by CSE. hTERT-ASM or primary ASM cells were treated for 4 and 24 hrs with 15% CSE. Then, cells were lysed for protein and mRNA determination. mRNA (A) and protein expression (C) of Epac1 was significant down regulated after exposure to CSE, while Epac2 was unaffected in hTERT cells. Same results were obtained in primary cells (E) Catalytic (PKA-C) and regulatory type II (PKA-RII) subunits of PKA mRNA (B & F) and protein (D) expression were not affected by CSE exposure in hTERT cells and in primary ASM cells. Protein expression of Epac1 and Epac2 (C) and PKA-C PKA-RII (D) were normalized to β-actin (for Epac) and GAPDH (for PKA). mRNA expression was normalized to 18 S. Data represent mean±SEM of 3–5 independent experiments. Statistical analysis was performed by one-way ANOVA followed by a Newman-Keuls post-hoc test. ^*^ *P*<0.05 compared to time point 0 hrs.

CSE down-regulated Epac1 mRNA and protein expression (Fig. 6A & C; *P*<0.05 both) in hTERT-ASM cells after 24 hrs of stimulation, leaving the mRNA and protein expression of Epac2 (Fig. 6A & C) and of the two subunits of PKA (Fig. 6B & D) unaffected. In *primary* human ASM, CSE-induced down-regulation of Epac1 mRNA was already apparent at 4 hrs after CSE treatment (Fig. 6E) and remained down regulated after 24 hrs (*P*<0.05 both). Similar to the findings in the hTERT-ASM cells, the expression of Epac2 and PKA mRNA was not significantly affected by CSE (Figs. 6F).

### Expression patterns of Epac and PKA in lung tissue from COPD patients

**Figure 7:**
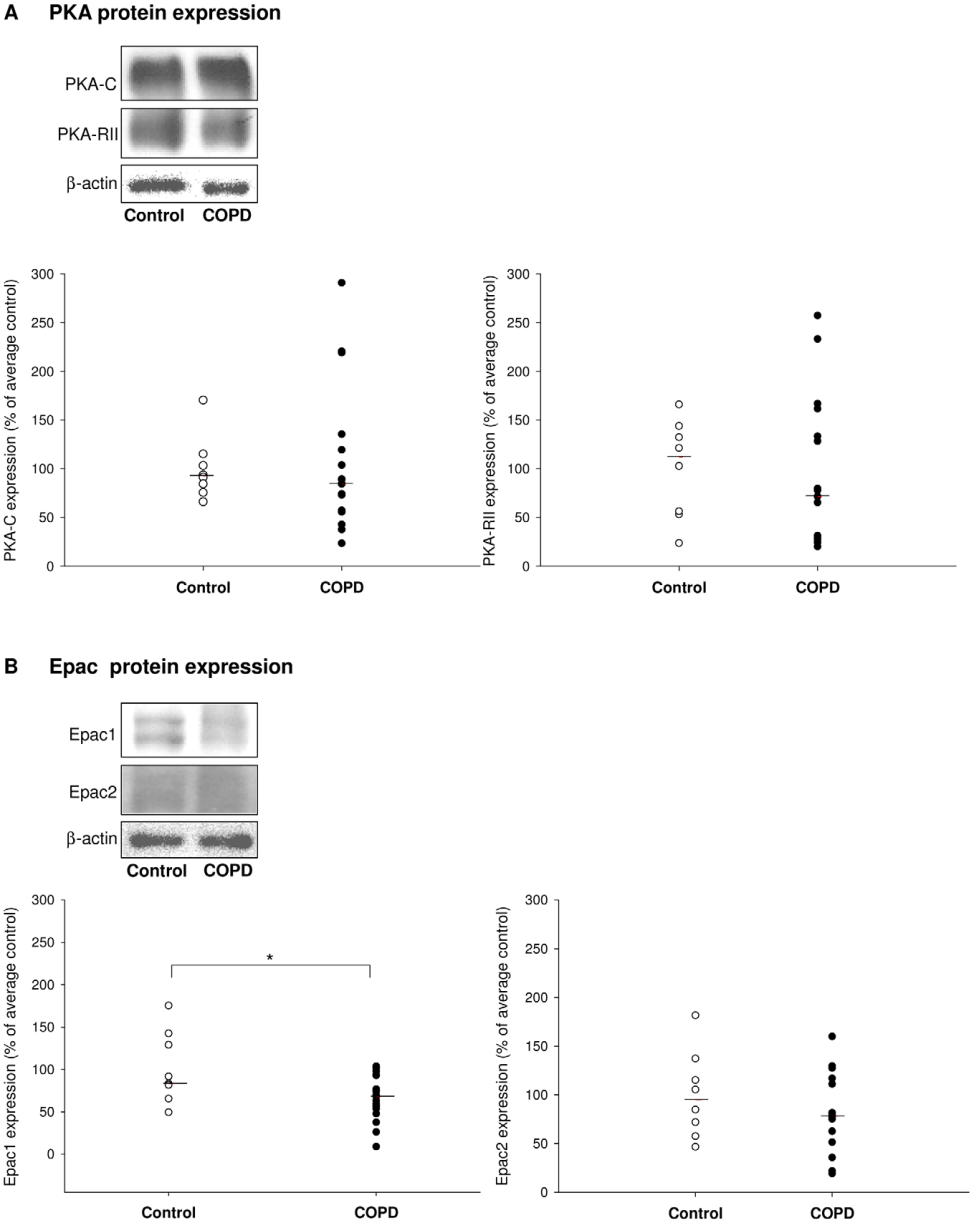
Epac and PKA expression in COPD patients. Expression of PKA-C and PKA-RII (A) and Epac1 and Epac2 (B) was evaluated by immunoblotting. Equal protein loading was verified by the analysis of β-actin. Responses were quantified by densitometry and normalized to the expression of β-actin. Data are derived from 9 controls and 15–19 COPD patients. Median of each group is indicated by -----. *P<0.05. Statistical differences between control and COPD were determined by non-parametric Mann-Whitney test.

Given the effect of CSE on cultured human ASM cells, we also evaluated the expression of Epac and PKA in lung tissue from COPD patients and from asymptomatic smokers, whose characteristics are described in Table 1. In line with the data in human ASM cells (Fig. 6), no change was observed in the protein (Fig. 7A–B) and mRNA expression (Figure S1) of Epac2 and PKA. Although no differences were observed for Epac1 mRNA (Figure S1), the expression of Epac1 protein was significantly lower in COPD patients (*P*<0.05) (Fig. 7B).

**Table 1:** Characteristics of the study subjects.

	Control	COPD
**Number of subjects**	9	20
**Age (years)**	62 (46–78)	60.5 (44–81)
**Male/female**	3/6	12/8
**Ex-smoker/current smoker**	7/2	16/4
**Pack years**	30.0 (3.0–52.5)	31.5 (8.5–65.0)
**FEV1% predicted**	96.9 (71.9–134.0)	37.8 (14.0–75.8)***
**FEV1/FVC %**	72.7 (70.6–85.3)	43.7 (19.2–71.1)***

All values except number of subjects, gender and smoking status are expressed as median values with minimum and maximum range in parentheses. Ex-smoker: stopped smoking for at least one year. FEV1% predicted: forced expiratory volume in 1 second as percentage of predicted value. FVC: forced vital capacity.

***P<0.001 compared to control group.

## Discussion

In this study, we show for the first time that the cAMP effectors Epac and PKA exert a distinct inhibitory role on CSE-induced release of IL-8 from human ASM cells. Thus, it was demonstrated that Epac activation inhibits CSE-induced IL-8 release by blocking NF-κB activation, whereas activation of PKA inhibits ERK activation. Moreover, CSE significantly reduces Epac1 expression in primary and immortalized human ASM cells, leaving Epac2 and PKA unaffected. Importantly, Epac1 protein abundance is also significantly reduced in COPD patients, which may translate our *in vitro* findings to a pathophysiological context.

Cigarette smoke contributes to the development of COPD by inducing a chronic inflammation known to be associated with irreversible damage of the airways and lung parenchyma [1], [34], [35]. The observed pathogenetic potential of cigarette smoke correlates in part with the increased release of the neutrophil chemoattractant IL-8 by inflammatory and structural cells [6], [7], [11]–[13] such as ASM cells [14], [15]. Current objectives of COPD therapy are to reduce episodes of airway obstruction and improve airflow limitation as a means of improving quality of life. Currently, no treatment effectively inhibits inflammation-driven progressive decline in lung function [36], although recently, some positive effects of long term corticosteroids have been observed [37]. Hence, there is a need for novel targets of anti-inflammatory therapy in this disease.

Beside its beneficial acute bronchodilatory effects, cAMP also exhibits *in vitro* anti-inflammatory properties in several cell types, by inhibiting the release of cytokines by several cell types in the airways [24]–[26]. This effect has typically been associated with activation of PKA [26]. However, our prior data have shown that next to PKA the novel cAMP effector Epac modulates bradykinin-induced IL-8 release from human ASM cells [31]. Only few studies have addressed the role of cAMP in CSE-induced IL-8 release [11], [38], and the effects of both Epac and PKA on this response have not been investigated at all. CSE-induced IL-8 release by human neutrophils is decreased by the β_2_-agonist salmeterol [11], but it only reduces IL-8 release by human ASM cells in the presence of fluticasone in studies using human ASM cells [38]. In contrast, here we report that the β_2_-agonist fenoterol alone can reduce CSE-induced IL-8 release from human ASM cells by itself, an effect that is mimicked by specific activation of Epac and PKA. Differences in conditions for cell culture (i.e. high and low passage tracheal vs bronchial *primary* ASM cells) and treatment (salmeterol vs fenoterol and incubation time) could account for the different effects observed. Importantly, our current study definitively demonstrates the capacity for both Epac and PKA to regulate CSE-induced inflammatory cytokine release in immortalized and primary human ASM cells.

Epac and PKA appear to have similar effects with regard to ASM synthetic, proliferative and contractile capacities [31], [39], [40]. As no cross-inhibition of both cAMP effectors was observed, we can exclude a main contribution of cooperation between Epac and PKA in our findings. Interestingly, our results indicate that Epac and PKA exert their inhibitory effects towards CSE-induced IL-8 release via distinct signalling routes at the transcriptional level (as shown by induction of IL-8 mRNA). It has been shown that CSE activates both NF-κB and ERK to increase IL-8 release [11], [12], [14]. In the current study, we demonstrate that activation of Epac specifically inhibited CSE-induced NF-κB activation, whereas activation of PKA specifically reduced ERK phosphorylation. Thus, our study unravels novel distinct, but complementary, immunosuppressive mechanisms of Epac and PKA in human ASM. Here we define NF-κB as the main target for the anti-inflammatory role of Epac, whereas the anti-inflammatory effect of PKA relies on ERK.

Despite the fact that both Epac and PKA are expressed in cultured primary and immortalized human ASM cells [31], [39] and reduce CSE-induced IL-8 release, by using effective concentrations of their selective activators we observed a stronger inhibitory effect of PKA compared to Epac. The lower inhibitory efficacy of the Epac activator 8-pCPT-2′-*O*-Me-cAMP towards CSE may relate to the observed reduced Epac1 abundance after CSE treatment. As specific inhibitors of Epac are currently not available, we tested the effects of Epac1 and 2 co-silencing on CSE-induced IL-8 release by using the siRNA technique. This technique reduced Epac mRNA and protein levels, in agreement with our previous data [31]. Such a diminishment of Epac expression resulted in a similar reduction of the anti-inflammatory effects of Epac activation. This demonstrates that both isoforms of Epac are implicated in the reduction of CSE-induced IL-8 release.

Importantly, we translated our findings to a pathophysiological setting showing reduced Epac1 expression in lung homogenates from COPD patients. Interestingly, our data indicate that Epac1 down-regulation is not solely related to acute smoking as it persists after smoking cessation in COPD patients. Our data point at post-translational modification of Epac1, as Epac1 mRNA levels in COPD patients did not differ from the mRNA levels in the control subjects. Lung damage and chronic inflammation by cigarette smoke-induced oxidative stress caused irreversible alterations in protein structure [41]. Such alterations might play a role in the down-regulation of Epac1 protein in COPD patients. Reduced expression of Epac1 has previously been shown in human lung fibroblasts by the action of the COPD-associated pro-fibrotic factor transforming growth factor-β [42].

These findings might have important clinical implications towards a better understanding of COPD pathogenesis and the improvement of its pharmacological treatment. Indeed, despite its anti-inflammatory effects *in vitro*, clinical studies only show modest beneficial effects of β_2_-agonists in the treatment of airway inflammation [43], [44]. Such a discrepancy has been assigned to variable β_2_-adrenergic receptor abundance or its potential polymorphisms [45] and additionally to alterations of β_2_-adrenergic receptor signalling by homologous or heterologous desensitization in both inflammatory and structural cells in the airways [28], [46], [47]. As a potential effector in cAMP-driven and β_2_-adrenergic receptor-induced signalling and a newly discovered inhibitor of NF-κB-dependent inflammatory response, Epac1 down-regulation by cigarette smoke may provide an additional explanation for the variable anti-inflammatory capacities of β_2_-agonists in the treatment of COPD.

In conclusion, Epac and PKA inhibit CSE-induced IL-8 release by human ASM by preventing the activation of NF-κB and ERK, respectively. These findings indicate that Epac and PKA might have potential as targets for anti-inflammatory therapy in COPD. Moreover, CSE-induced reduction in Epac1 expression might point to a divergent contribution of cAMP effectors in mediating immunosuppressive effects of current drug therapies. Hence, studying the mechanisms by which cigarette smoke exposure drives Epac1 down-regulation could unveil alternative ways for intervention, as targeting specific cAMP effectors could allow a more effective control of cAMP signalling driven by G_s_-coupled receptors.

## Methods

### Human lung tissue

Human lung tissue was collected from COPD patients and asymptomatic smokers, the latter as a control group (Table 1). Classification of COPD severity was based on the Global Initiative for Chronic Obstructive Lung Disease (GOLD) criteria [36]. Tissue from the control group and from GOLD stage II patients was derived from noninvolved lung tissue of patients undergoing resective surgery for pulmonary carcinoma; the control group had no airway obstruction or chronic airway symptoms, such as cough and sputum production. Tissue from GOLD stage IV patients was collected from subjects undergoing surgery for lung transplantation. The COPD-patients did not have clinical signs of chronic bronchitis and were not suffering from alpha-1-antitrypsin deficiency. The clinical characteristics of the patients are given in Table 1. The study protocol was consistent with the Research Code of the University Medical Center Groningen (http://www.rug.nl/umcg/onderzoek/researchcode/index) and national ethical and professional guidelines (“Code of conduct; Dutch federation of biomedical scientific societies”; htttp://www.federa.org).

### Cigarette smoke extract

Cigarette smoke extract was prepared as previously described [33]. Briefly, 2 research cigarettes (University of Kentucky 2R4F; filters removed) were combusted using a peristaltic pump (Watson Marlow 323 E/D, Rotterdam, The Netherlands) and passing the smoke through 25 ml of FBS-free DMEM supplemented with antibiotics at a rate of 5 minutes/cigarette. The solution obtained is referred to as 100% strength and was then diluted in FBS-free DMEM+antibiotics to obtain a 15% working solution.

### Cell culture

Human bronchial smooth muscle cell lines, immortalized by stable ectopic expression of human telomerase reverse transcriptase enzyme (hTERT-ASM), passages 1–30 were used. All procedures were approved by the human Research Ethics Board of the University of Manitoba [48]. Primary human ASM cells were isolated from human tracheal sections from anonymized lung transplantation donors (obtained from the Department of Cardiothoracic Surgery, University Medical Center Groningen) as previously described [39]. Passages 1–5 were used. Prior to the experiments, cells were growth arrested overnight and treated with fenoterol, 8-pCPT-2′-*O*-Me-cAMP or 6-Bnz-cAMP for 20 min before stimulation with CSE. The PKA inhibitors (H89, Rp-8-Br-cAMPS plus Rp-cAMPS), the MEK inhibitor U0126 and the IKK-2 inhibitor SC514 were added 30 min before the other stimuli [40]. An Alamar blue assay was used to determine cell viability as described previously [31].

### IL-8 assay

24 hrs after stimulation of ASM cells with CSE, culture medium was collected for the determination of IL-8 concentrations by ELISA according to the manufacturer's instructions (Sanquin, the Netherlands), as previously described [31].

### Silencing of Epac1 and Epac2 expression

Epac1 and Epac2 knock-down was achieved by transfection of hTERT-ASM cells (90% confluency) with 200 pmol of siRNA (Table S1). ON-TARGET plus Non-targeting Pool (D-001810-10-20) was used as a negative control (control siRNA). Lipofectamine 2000 (1 mg/ml) was used as a vehicle. 6 hrs after transfection, cells were washed. The day after, cells were seeded in 24 wells plates and serum-deprived overnight. 24 hrs after stimulation with CSE in the absence or presence of 8-pCPT-2′-*O*-Me-cAMP or 6-Bnz-cAMP, supernatants were collected for analysis of IL-8 levels. Furthermore, transfected cells were lysed for analysis of Epac1 and Epac2 protein or mRNA.

### Western analysis

Cell lysates were prepared and subjected to protein determination using a Pierce BSA protein assay (Thermo Scientific, Rockford, IL, USA). Similar protein amounts were loaded on a SDS-Page gel (8–15%) for electrophoresis and transferred to a nitrocellulose membrane. Incubation with the specific primary and secondary antibodies was performed overnight (Table S2). After the addition of western lighting plus-ECL, determination of protein bands was achieved using the G-BOX iChemi (Syngene, Cambridge, UK). Bands were normalized to GAPDH, β-actin or ERK, depending on the protein under investigation.

### mRNA isolation and RT-PCR

mRNA from cultured human ASM cells was extracted by using a Nucleospin RNA II kit (Machery Nagel, Düren, Germany) and quantified using spectrophotometry (Nanodrop, ThermoScientific, Wilmington, USA). 1 µg of mRNA was converted in cDNA by reverse transcriptase using Promega tools (Madison, WI, USA). cDNA was subjected to real-time PCR (RT-PCR) using a MyiQ™ Single-Color detection system (Bio-Rad Laboratories Inc. Life Science Group, Hercules, CA, USA) and specific primers (Table S3). The amount of target gene was normalized to ribosomal subunit 18 S (designated as ΔCT). Relative differences were determined using the equation 2^−(ΔΔCt)^. When necessary, a 1.2% gel was used to separate the RT-PCR products.

### Immunofluorescence

10.000 cells per well were plated in coverslips and serum deprived for 24 hrs before stimulation. Cells were fixed by addition of 3% paraformaldehyde (PFA) for 15 min, followed by incubation in 3% PFA plus 0.3% Triton X-100 for 5 min. After blocking, cells were incubated overnight with p65 antibody (Santa Cruz, CA, USA, dilution 1∶20) and donkey-anti rabbit FITC (dilution: 1∶50) was added. Nuclei were stained with a Hoechst staining (1∶10000). After this, coverslips were mounted using ProLong Gold antifade reagent and analyzed using an Olympus AX70 microscope equipped with digital image capture system (ColorView Soft System with Olympus U CMAD2 lens).

### Materials

6-Bnz-cAMP and 8-pCPT-2′-*O*-Me-cAMP and the PKA inhibitors Rp-cAMPS and Rp-8-Br-cAMPS were purchased from BIOLOG Life Science Institute (Bremen, Germany). Fenoterol was obtained from Boehringer Ingelheim (Ingelheim, Germany). Protease inhibitors, albumin bovine serum, Triton X-100, H89 were from Sigma-Aldrich (St-Louis, MO, USA). Cell medium components were from GIBCO-BRL Life Technologies (Paisley, UK). Donkey serum and donkey anti-rabbit FITC fluorescent antibody was obtained from Jackson Immuno Research (West Grove, PA, USA). Hoechst nuclear staining, anti-fade solution, lipofectamine 2000 (1 mg/ml) and Alamar Blue were purchased from InVitrogen (Carlsbad, CA, USA) as well as the PCR primers. ECL solution for western blot detection was purchased from PerkinElmer Inc. (Waltman, MA, USA). siRNA probes were obtained from Dharmacon Inc. (Lafayette, CO, USA). U0126 was from Tocris Cookson Inc. (Bristol, UK). SC514 was obtained from Calbiochem (Amsterdam, The Netherlands).

### Statistical analysis

For cell culture experiments with multiple comparisons results are expressed as mean±SEM of separate experiments and one-way ANOVA followed by the indicated post hoc tests, was performed.

For data derived from COPD and control subjects, values represent medians (range between parenthesis) and a non-parametric Mann Whitney test was used to evaluate differences. *P* values<0.05 were considered to be statistically significant.

## Supporting Information

Figure S1 Epac and PKA mRNA expression in COPD patients. Expression of PKA-C and PKA-RII (A) and Epac1 and Epac2 (B) was evaluated by qRT-PCR. Values were relative to 18 S values. Data are derived from 9 controls and 15–19 COPD patients. Median of each group is indicated by -----. Statistical differences between control and COPD were determined by non-parametric Mann-Whitney test. No statistical differences were found.
(TIF)

Table S1 Sequences of siRNA probes against Epac1 and Epac2.
(DOCX)

Table S2 List of antibodies used in western analysis.
(DOCX)

Table S3 Primers used for RT-PCR.
(DOCX)
