# Supplementary figures and images for: Anti-Inflammatory Role of the cAMP Effectors Epac and PKA: Implications in Chronic Obstructive Pulmonary Disease
Source: PLoS One. 2012 Feb 21;7(2):e31574. doi: 10.1371/journal.pone.0031574 (PMC3283666; doi:10.1371/journal.pone.0031574)

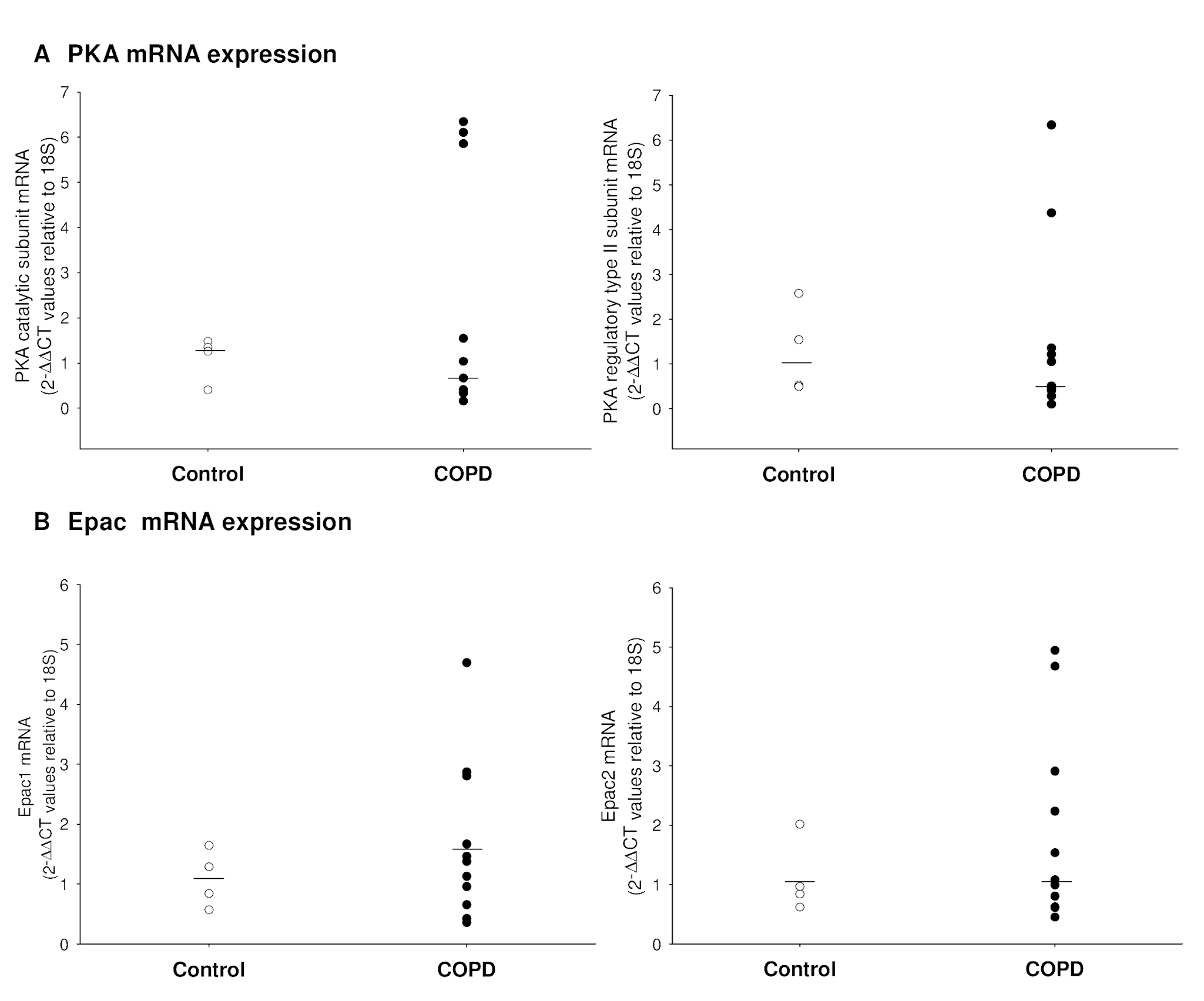

Supplement: Figure S1 — Epac and PKA mRNA expression in COPD patients. Expression of PKA-C and PKA-RII (A) and Epac1 and Epac2 (B) was evaluated by qRT-PCR. Values were relative to 18 S values. Data are derived from 9 controls and 15–19 COPD patients. Median of each group is indicated by -----. Statistical differences between control and COPD were determined by non-parametric Mann-Whitney test. No statistical differences were found. (TIF) [file pone.0031574.s001.tif]
