# Supplementary material for: Anti-Inflammatory Role of the cAMP Effectors Epac and PKA: Implications in Chronic Obstructive Pulmonary Disease
Source: PLoS One. 2012 Feb 21;7(2):e31574. doi: 10.1371/journal.pone.0031574 (PMC3283666; doi:10.1371/journal.pone.0031574)
Supplement: Table S1 — Sequences of siRNA probes against Epac1 and Epac2. (DOCX) [file pone.0031574.s002.docx]

**Table S1**. Sequences of siRNA probes against Epac1 and Epac2.

| **Epac1** | **ON-TARGETplus SMART pool siRNA** | **Target sequence (5'-3')** |
| --- | --- | --- |
|  | J-007676-05 | CGUGGGAACUCAUGAGAUG |
|  | J-007676-06 | GGACCGAGAUGCCCAAUUC |
|  | J-007676-07 | GAGCGUCUCUUUGUUGUCA |
|  | J-007676-08 | CGUGGUACAUUAUCUGGAA |
| **Epac2** | **ON-TARGETplus SMART pool siRNA** | **Target sequence (5'-3')** |
|  | J-009511-05 | GAACACACCUCUCAUUGAA |
|  | J-009511-06 | GGAGAAAUAUCGACAGUAU |
|  | J-009511-07 | GCUCAAACCUAAUGAUGUU |
|  | J-009511-08 | CAAGUUAGCACUAGUGAAU |
