# Supplementary material for: Anti-Inflammatory Role of the cAMP Effectors Epac and PKA: Implications in Chronic Obstructive Pulmonary Disease
Source: PLoS One. 2012 Feb 21;7(2):e31574. doi: 10.1371/journal.pone.0031574 (PMC3283666; doi:10.1371/journal.pone.0031574)
Supplement: Table S2 — List of antibodies used in western analysis. (DOCX) [file pone.0031574.s003.docx]

**Table S2**. List of antibodies used in western analysis.

| **Target** | **Source (catalog number)** | **Dilution** | **Secondary antibody**  **(dilution 1:2000)** |
| --- | --- | --- | --- |
| VASP | Cell Signalling (3112) | 1:500 | rabbit |
| p-ERK | Cell Signalling (9101S) | 1:1000 | rabbit |
| ERK | Cell Signalling (9102) | 1:500 | rabbit |
| Epac1 | Cell Signalling (41555) | 1:500 | mouse |
| Epac2 | Cell Signalling (41565) | 1:500 | mouse |
| PKA-C | BD Transduction Laboratories  (610980) | 1:500 | mouse |
| PKA-RII | BD Transduction Laboratories  (558244) | 1:500 | mouse |
| IkBα | Santa Cruz (sc-203) | 1:200 | rabbit |
| GAPDH | Santa Cruz (sc-47724) | 1:2000 | mouse |
| β-actin | Sigma (A 5441) | 1:2000 | mouse |
