# Supplementary material for: Anti-Inflammatory Role of the cAMP Effectors Epac and PKA: Implications in Chronic Obstructive Pulmonary Disease
Source: PLoS One. 2012 Feb 21;7(2):e31574. doi: 10.1371/journal.pone.0031574 (PMC3283666; doi:10.1371/journal.pone.0031574)
Supplement: Table S3 — Primers used for RT-PCR. (DOCX) [file pone.0031574.s004.docx]

**Table S3**. Primers used for RT-PCR.

| **Primers** |  | **Sequence 5'-3'** |
| --- | --- | --- |
| Epac1 | Forward | GACCGAGATGCCCAATTCTA |
| Epac1 | Reverse | TTTCGAAGTGCCACAAGTGAG |
| Epac1 | Forward | GCGTAATACGACTCACTATAGGGAGAGATGGGACTTCTCCCTCCTC |
| Epac1 | Reverse | GCGTAATACGACTCACTATAGGGAGACTGCTTGACCTCCTTTCAGG |
| Epac2 | Forward | ATTGCAAATACGGCCAGAAC |
| Epac2 | Reverse | TCTATGGTCGACGAGGCTCT |
| IL-8 | Forward | TAGCA AATTGAGGCCAAGG |
| lL-8 | Reverse | AAACCAAGGCACAGTGGAAC |
| Ribosomal subunit 18S | Forward | CGCCGCTAGAGGTGAAATTC |
| Ribosomal subunit 18S | Reverse | TTGGCAAATGCTTTCGCTC |
